# Plasmonic and silicon spherical nanoparticle antireflective coatings

**DOI:** 10.1038/srep22136

**Published:** 2016-03-01

**Authors:** K. V. Baryshnikova, M. I. Petrov, V. E. Babicheva, P. A. Belov

**Affiliations:** 1 ITMO University, Kronverkskiy, 49, St. Petersburg 197101, Russia; 2 St. Petersburg Academic University RAS, Khlopina 8/3, 194021, Saint-Petersburg, Russia; 3 Center for Nano-Optics, Georgia State University, P.O. Box 3965, Atlanta, GA 30302, USA.

## Abstract

Over the last decade, plasmonic antireflecting nanostructures have been extensively studied to be utilized in various optical and optoelectronic systems such as lenses, solar cells, photodetectors, and others. The growing interest to all-dielectric photonics as an alternative optical technology along with plasmonics motivates us to compare antireflective properties of plasmonic and all-dielectric nanoparticle coatings based on silver and crystalline silicon respectively. Our simulation results for spherical nanoparticles array on top of amorphous silicon show that both silicon and silver coatings demonstrate strong antireflective properties in the visible spectral range. For the first time, we show that zero reflectance from the structure with silicon coatings originates from the destructive interference of electric- and magnetic-dipole responses of nanoparticle array with the wave reflected from the substrate, and we refer to this reflection suppression as substrate-mediated Kerker effect. We theoretically compare the silicon and silver coating effectiveness for the thin-film photovoltaic applications. Silver nanoparticles can be more efficient, enabling up to 30% increase of the overall absorbance in semiconductor layer. Nevertheless, silicon coatings allow up to 64% absorbance increase in the narrow band spectral range because of the substrate-mediated Kerker effect, and band position can be effectively tuned by varying the nanoparticles sizes.

The suppressing of light reflection from a flat surface has been an important technological problem for the last decades. The methods of canceling reflection rely on utilizing different optical elements starting from a simple quarter-wavelength dielectric layer, to nanostructured surfaces for light trapping, graded-index layers, and others^1^,^2^. Recently plasmonic nanostructures have been shown to possess a lot of advantages^3^, most of which are related to excitation of the intense localized surface plasmon resonance (LSPR) in metallic nanostructures and strong suppression of light reflection in wavelength region close to the resonance^4^. Despite the active studies on these topics and possible large impact of the plasmonic applications, practical use of plasmonic nanoparticles is still hindered by many challenges, for instance, large ohmic losses of metals^5^, which suppress nanoparticle resonances, or parasitic surface oxidizing, which changes the optical properties of nanostructures^6^. Tuning the optical properties of plasmonic nanostructures can be realized through changing the nanoparticle shape or interparticle distances rather than the nanoparticle characteristic dimensions^7^. Recently the all-dielectric photonics emerged as a promising alternative to plasmonics^1^,^8^,^9^. Its concept is based on designing of high refractive index nanostructures, which possess magnetic Mie resonance along with electric one and allow simultaneous control of magnetic and electric components of light on the nanoscale^10^. Silicon is considered as one of the most suited materials for all-dielectric photonics having high refractive index and relatively low optical losses in the visible and near-infrared wavelength ranges^11^. Resonant spectra of high-index structures are defined by their characteristic dimensions together with optical properties of bulk material and consequently can be efficiently tuned during the fabrication process^12^. Furthermore, researchers’ attention has recently been attracted to important features of high-index nanoparticles: at a certain wavelength they possess a high directionality of radiation pattern^13^,^14^, which results in strong forward and low backward scatterings. This behavior was predicted for particles with equal magnetic and electric dipole moments by Kerker and colleagues^15^, and such particles are often referred as Huygens element^16^,^17^,^18^. Huygens elements are proposed to be used as main functional element of metasurfaces and future flat photonic devices for efficient light manipulations on the nanoscale^19^.

Plasmonic nanostructures have already shown their capability of solar cells efficiency improving, and both light direction guidance (far-field) and light concentration (near-field) were experimentally demonstrated to enable photocurrent increase and higher efficiency of about 8–40%^20^. With regard to the antireflection properties of plasmonic coatings, number of experimental studies have demonstrated a sharp dip in reflection spectrum of silver nanoparticles coating for wavelength slightly larger than wavelength of LSPR^21^,^22^. Recently, the antireflectance properties of emerged all-dielectric nanostructures have been studied showing the tunability of silicon nanopillar-based structures^23^,^24^, which allows to construct broadband antireflection coatings for photovoltaics systems^25^. It is worth noting that in the recent paper^26^ narrow-band antireflection properties of silicon spherical particles on a high-index substrate were shown using single nanoparticle microspectroscopy, and here we analyze this effect in more detail.

In this manuscript, we are aiming on utilizing the controllable scattering directivity of all-dielectric coatings for suppressing light reflection from silicon substrate and comparing their efficiency to plasmonic coatings. We chose nanoparticles of silver and silicon as the most favorable plasmonic and all-dielectric photonics materials, respectively. We clarify the physical background of antireflective properties of such nanoparticle arrays, and more importantly, show two different characters of nanoparticle interaction with high-index substrate that take place for either silver or silicon antireflective coating. In particular, we demonstrate that the antireflectance effect of silicon coatings originates from destructive interference of wave reflected from the substrate with the fields reradiated by the electric and magnetic dipoles induced in silicon nanoparticles. This effect can be considered as substrate-mediated Kerker effect as an analogue of well-studied Kerker effect for dielectric nanoparticles in air or homogeneous environment (low-index substrates and matched-index covering of nanoparticle array). This essentially differs from the case of plasmonic coatings, for which the antireflectance is related to strong interaction of plasmonic particle with the substrate (substrate-induced bi-anisotropy). Finally, we show high efficiency of light harvesting with silver and silicon coatings being placed on top of thin-film photovoltaic elements. We numerically calculated the light absorption inside photovoltaic elements in both broad and narrow bands and discuss the efficiency of the coatings comparing to each other and to uncoated structure.

## Results

### Structure model and simulation parameters

According to the Mie theory, single silicon particles possess dielectric-type dipole resonances at the wavelength λ fulfilling the condition 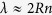, where *R* is the nanoparticle radius and *n* is the real part of the silicon refractive index^11^. Silicon particles with radius 40–80 nm have only electric (ED) and magnetic dipole (MD) resonances in the visible spectral range, and the higher multipole modes are weak and is in the ultra-violet range^27^. Because of the high refractive index of silicon in the spectral range under consideration, we use term “dielectric” for silicon nanoparticles and nanostructure, even though they are semiconducting. Though this term might be confusing, this terminology is generally accepted in the literature^28^,^29^.

Silver particles support LSPR of the dipole mode, which is defined mainly by the permittivities of metal 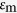 and surrounding material 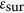. Particularly, for dipole LSPR in spherical nanoparticles, which is small in comparison to wavelength, permittivities should satisfy the condition 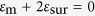, which in case of silver in air occurs in the ultra-violet range^5^.

**Figure 1:**
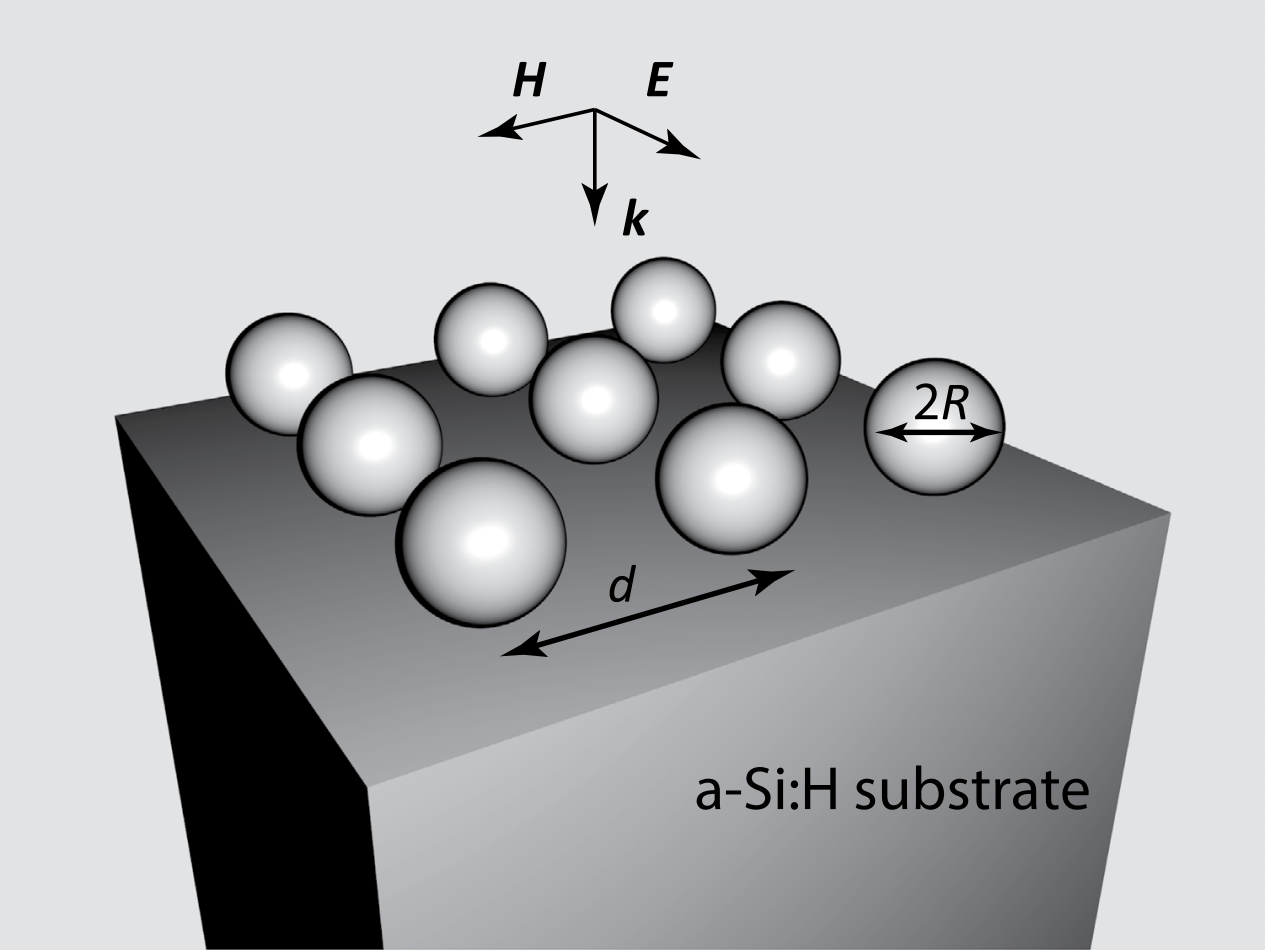
Square periodic array of spherical nanoparticles (out of either silver or silicon) with radius *R* and period *d* on top of a-Si:H substrate. The direction of incident wave is shown by the wave vector *k*.

We consider a square array of crystalline silicon (c-Si) and silver spherical nanoparticles (see Fig. 1) placed on top of hydrogenated amorphous silicon substrate (a-Si:H), which is widely-used material in thin-film solar cells and photodetectors^30^. The analysis of nanoparticle coatings for thin-film photovoltaic elements with p-i-n silicon layers is performed in the last section. To compare the efficiency of crystalline silicon and silver nanoparticle arrays for transmission enhancement, we restrict ourselves to the case of normal incidence of light and consider the particles of 15–80 nm radius. In this paper, all results are obtained for the fixed value of surface filling factor 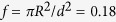, where *d* is a period of the array, making possible comparing structures with different nanoparticles size and keeping the dipole-dipole interaction of neighboring nanoparticles rather weak and constant for each coating material. Numerical simulations and postprocessing were performed in CST Microwave Studio and Comsol Multiphysics software packages. The permittivities of silver and silicon materials were taken from the previously obtained experimental measurements^5^,^30^,^31^.

### Substrate-mediated zero backscattering from nanoparticle array

**Figure 2:**
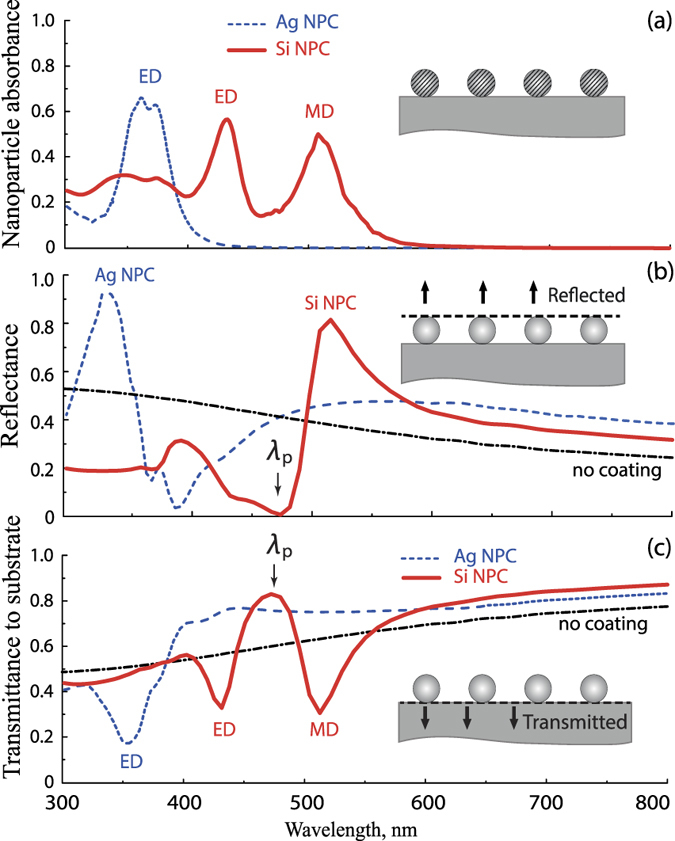
(**a**) Absorbance inside silicon and silver nanoparticles. Inset: nanoparticle array; particles are shaded to show the region of absorbance calculations. (**b**) Spectral dependences of the reflectance for bare substrate, for silver, and silicon coatings. Inset: nanoparticle array; dashed line right on top of nanoparticles shows where calculations were done. The wavelength of resonant transmission and suppression of reflection for Si-NPC is denoted as λ_*p*_. (**c**) Transmittance through the silicon surface without coating (dash-dot black line), or with silicon- (solid red line) and silver- NPC (dashed blue line). Inset: nanoparticle array; dashed line right beneath nanoparticles shows the place of calculation of transmittance in the direction shown by the arrows (losses inside the silicon substrate do not directly affect transmitted wave). The calculations in (**a–c**) were performed for Ag-NPC *R*_Ag_ = 30 nm, *d*_Ag_ = 125 nm and for Si-NPC *R*_Si_ = 60 nm, *d*_Si_ = 250 nm.

In order to identify the nanoparticle resonances, we have calculated the absorbance inside the nanoparticles. The particular set of parameters: for silicon nanoparticle coating (Si-NPC) *R*_Si_ = 60 nm and *d*_Si_ = 250 nm, and for silver nanoparticle coatings (Ag-NPC) *R*_Ag_ = 30 nm and *d*_Ag_ = 125 nm, provides conditions for the excitation of all resonances in the range of silicon-photovoltaics operation^25^,^32^, which overlaps with the range 300 nm < λ < 800 nm considered through the paper. In the following subsection we show and discuss results for other parameters of NPCs, which resonances also fall within this range. The absorbance inside the nanoparticles shows spectral positions of nanoparticle resonant modes [Fig. 2(a)]: the LSPR for metal nanoparticles (labeled “ED”) and two Mie-resonances for silicon nanoparticles – electric at shorter wavelength (labeled “ED”) and magnetic at longer wavelength (labeled “MD”). For the chosen radiuses, absorption resonances of silicon and silver nanoparticles have similar widths and comparable heights. Spectral position of the LSPR in silver nanoparticles is in a good agreement with resonance condition for spherical nanoparticle in air 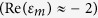, and the slight splitting of the peak results from the interaction of the nanoparticle with the high-index substrate and induced bianisotropy effects^33^, which we discuss below in more detail.

Now, let us analyze the reflectance and transmittance spectra of nanoparticle arrays and influence of high-index substrate on the blooming effect [Fig. 2(b,c)]. The calculated reflectance spectra of Ag-NPC and Si-NPC demonstrate narrow-band strong antireflectance properties, which manifest themselves in sharp decreases of reflectance at the wavelength 400 nm for Ag-NPC (decreasing of reflectance from 47% for uncoated silicon to 3% with coatings) and 480 nm for Si-NPC (decreasing of reflectance from 39% for uncoated silicon to 0.4% with coating) [Fig. 2(b)]. From the plot, we see that for silicon coating, the antireflectance effect occurs spectrally between the ED and MD resonances, which are known to govern its optical properties. The interplay of magnetic and electric dipole resonant modes in dielectric nanostructures was experimentally observed, for example, through the asymmetry of light scattering by individual silicon nanoparticles^13^. This feature is explained by Kerker effect^14^,^29^,^34^, which was predicted for optical elements with electric and magnetic dipole moments equal both in amplitudes and in phase. Such elements are often referred as Huygens elements^14^,^19^, as equality of electric and magnetic moments means that effective impedance of the metasurface [proportional to √(μ/ε)] is equal to the air impedance, which ensures transmittance through the array of elements in air without reflection^35^.

The strongly unidirectional scattering of Huygens elements and its drastic influence on the transmittance spectrum of the whole array provide the basis for engineering metasurfaces for flat nanophotonic devices. For instance, in the recent works, silicon nanodisks were studied in homogeneous low-index environment^19^,^35^, and the possibility of light phase change by simultaneous controlling of magnetic and electric dipole resonances was demonstrated. In fact, the vast majority of studies were performed for nanoparticles on top of low-index substrate or theoretically considering them in air. The detailed studies^36^ revealed that the Kerker effect, which manifest itself in high forward-to-backward ratio of scattering from a single nanoparticle, is significantly modified and partially suppressed by placing the nanoparticle over the substrate with high refractive index. Moreover, in that case, dipole-moment modes of the studied nanodisks suffer from leakage to the substrate^25^, which causes a decrease of total scattering.

**Figure 3:**
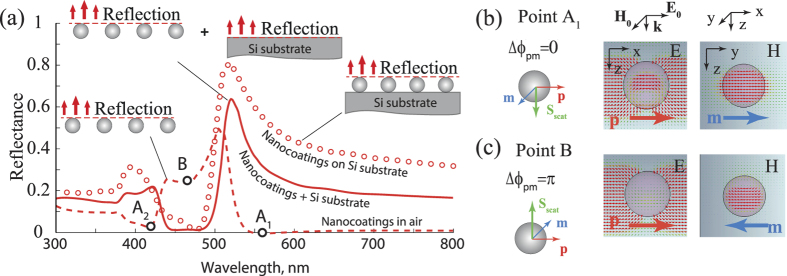
(**a**) Reflectance spectra for a plane wave normally incident over silicon nanoparticle array in air (dashed line) and on top of the silicon substrate (round circles). The solid line shows sum of the fields reflected from the nanoparticle array in air and from the bare silicon substrate. The parameters of the array are identical to the parameters in Fig. 2(b,c) The corresponding electric and magnetic field distributions define orientations of the electric **p** and magnetic **m** dipole moments in silicon nanoparticles array in air (unit cell is shown). Two wavelengths are shown: (**b**) point A_1_, corresponding to the Kerker effect in homogeneous environment, and (**c**) point B, corresponding the position of antireflectance of Si-NPC.

To clarify the role of magnetic and electric dipoles’ scattering in the antireflectance effect for the case of nanoparticle array on silicon substrate, we have performed additional numerical simulations and separated contributions of the nanoparticle coating and the substrate in the total reflectance. First, we have calculated the reflectance spectrum for a plane wave normally incident at the array of silicon nanoparticles in air (shown in Fig. 3(a) with dashed line). The zero-reflectance at point A_1_ at the long-wavelength side of the magnetic resonance and suppressed reflection at point A_2_ on the short-wavelength side of electric resonance correspond to Kerker effect (e.g. observed in^13^ and^14^ for similar nanoparticles). The electric and magnetic dipole moments are in-phase with each other, and equal in amplitude, which results in zero backscattering from the individual nanoparticles [Poynting vector **S**_scat_ points down, see Fig. 3(b)]. At the same time, between the ED and MD resonances, at the point B, one can see a high reflectance, which corresponds to strong backscattering because of almost 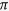-shift between the electric and magnetic dipole moments phases [Poynting vector **S**_scat_ points up, see Fig. 3(c)]. For the wavelength range between ED and MD resonances, the wave reflected from the nanoparticle array has π/2 phase shift relative to the incident wave, and has opposite direction. Second, we calculated the wave reflected from bare silicon substrate, and we have sum it up with the wave reflected from nanoparticle array in air. The wave reflected from the bare silicon substrate has π-phase shift relative to the incident wave at the plane of nanoparticle centers and additional 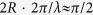 phase incursion. Thus, the wave directly reflected from the substrate has 3π/2 phase shift relative to the incident wave. After summing up with the wave scattered by the nanoparticle array (with π/2 phase)^19^, waves cancel out each other, which gives zero reflectance between ED and MD resonances (the resulting curve is shown in Fig. 3(a) with solid line). Comparing the obtained reflectance spectrum with the spectrum calculated for the Si-NPC over the silicon substrate, one can see a good quantitative agreement with our simple model. This model implies that spherical silicon nanoparticles weakly interact with the substrate, and one can consider Si-NPC and substrate as decoupled systems. To summarize, our calculations for the nanoparticle array on silicon substrate show that there exists a wavelength range with drastic decrease of reflectance (430–480 nm), which appear because of the destructive interference of magnetic and electric dipole scattered waves and the wave reflected from the substrate. In this sense, the predicted antireflectance can be defined as a substrate-mediated Kerker effect.

Further, let us discuss the origin of blooming effect with the array of plasmonic nanoparticles [see Fig. 2(b)], which have only electric dipole resonance. The antireflectance effect for small metal nanoparticle array has been recently clarified and associated with the substrate-induced bi-anisotropy^33^,^37^. The electric dipole moment of the nanoparticle induces charge distribution in the high-index silicon substrate in the so-called “hot spots” beneath nanoparticle. Though the single metal particle does not possess any intrinsic magnetic dipole moment, the overall charge distribution inside the particle and in the substrate hot spot has non-zero magnetic moment. In this case, the destructive interference of the field scattered by ED and substrate-induced MD moments and the wave reflected from the silicon substrate causes the blooming effect. Substrate-induced MD moment results in the increase of absorbance, which can be either spectrally overlapped with ED peak or slightly separated depending on how high refractive index of the substrate is. In our case of the silicon substrate, we observe a negligible splitting in the absorbance spectra in Fig. 2(a). One can mention that the good agreement between the simulation results and the simple interference model for the case of Si-NPC [Fig. 3(a)] also implies that in contrast to the case of Ag-NPC, the interaction of Si-NPC with the substrate is rather weak^25^. Thus, we can conclude that the contribution of substrate-induced bi-anisotropy to antireflectance effect is negligible in comparison to substrate-mediated Kerker effect.

To sum up, although the origin of antireflectance for the case of high-index substrate is similar to well-known Kerker effect, spectral position and destructively interfering waves are different. For silicon particles embedded in homogeneous optical environment, Kerker effect occurs either on the long-wavelength side of the MD resonance or on short-wavelength side of ED^13^, and in both cases, it is sensitive to indices of substrate and superstrate of the nanoparticle. In contrast, high-index substrate, which is the case of the present work, possesses sufficient reflection, the substrate-mediated Kerker-like effect manifests itself in the destructive interference of the ED and MD fields with the wave reflected from the substrate surface, and the blooming effect is observed between the ED and MD resonances.

### Transmission efficiency of plasmonic and silicon nanoparticle coatings

The antireflective properties discussed above can be applied to increase light absorption inside photovoltaic elements. Further, we calculate efficiency of NPC in terms of increased transmittance and show that both types of coating can provide better light trapping. Transmittance to the substrate is shown in Fig. 2(c) and is calculated right beneath the nanoparticle array as shown in the inset. Comparing Fig. 2(a,c), one can see that for metal nanoparticles, transmittance to the substrate decreases at the LSPR due to the strong absorption inside nanoparticles (reported earlier in *e.g*^38^). The same effect is observed for Si-NPC, where the absorption inside nanoparticles is resonantly increased at the wavelengths of ED and MD resonances. Thus, it is important to stress that ED or MD resonance itself does not bring increase of transmittance, and this wavelength cannot be used to increase efficiency of light harvesting. However, both Ag-NPC and Si-NPC enhance transmission to the substrate at the wavelengths longer than ED and MD resonances, respectively [Fig. 2(c)]. It originates from the effect of constructive interference of scattered light with incident wave. Indeed, for wavelengths longer than the resonant, dipole oscillations occur in-phase with incident light, while below the resonant wavelength, dipole oscillates with 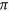-shift in respect to the driving field, which results in destructive interference observed for shorter wavelengths λ ≈ 300–350 nm for Si-NPC [see Fig. 2(c)].

To compare the antireflective efficiency of nanoparticle coatings of different nanoparticle radii in the range of 15–80 nm, we introduce integral transmission enhancement as follows:


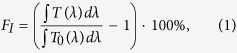


where *T*(λ) and *T*_0_(λ) are the transmittances calculated with and without coating, respectively; spectral integration is taken over the wavelength range from 300 to 800 nm.

**Figure 4:**
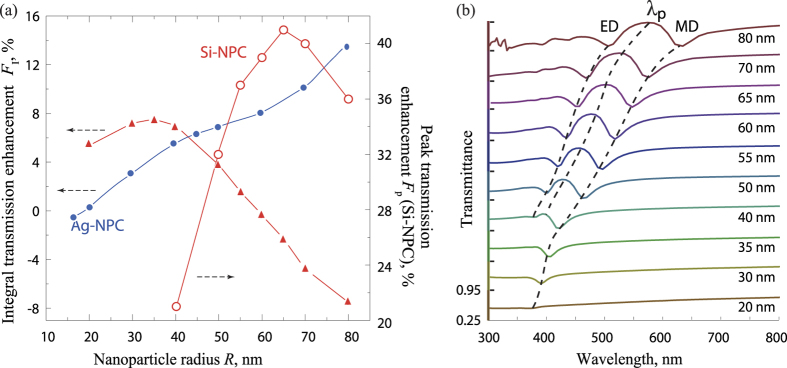
(**a**) Left axes: integral enhancement *F*_l_ for both types of coatings [Eq. (1)]. Right axes: the peak enhancement *F*_p_ [Eq. (2)] for Si-NPC at λ_*p*_. (**b**) Transmittance spectra for Si-NPC with nanoparticles of different radiuses *R* = 20–80 nm. The positions of transmission enhancement λ_*p*_, ED-, and MD-resonances are shown with the black dashed lines. On ordinate axis, each section is from 0.25 to 0.95, i.e. shifted on 0.7.

The dependence of the integral enhancement on the nanoparticle radius is plotted for different types of coatings in the Fig. 4(a). We see the non-monotonous dependence of the integral transmission enhancement for Si-NPC: the smaller nanoparticles show higher efficiency (up to 8%) than the larger ones, which occurs due to the stronger absorption inside the nanoparticles. The quadrupole response of the silicon nanoparticles with the sizes considered in this work is rather weak because of the high loss of silicon in ultra-violet range. For Ag-NPC, although particles with radius *R* < 20 nm demonstrate negative transmission efficiency *F*_l_, we see the increase of the efficiency with the increase of nanoparticle sizes: it is 8% for particles with *R* = 50–60 nm, and it reaches 15% for *R* = 80 nm. Here we should note that silver particles with the radius more than 30–40 nm demonstrate strong higher-order multipole response^38^. Thus, plasmonic and silicon nanoparticles of the same size demonstrate different optical properties: silver nanoparticles with large size allow multipolar-mode resonances, which increase integral transmission efficiency of the coating.

From practical point of view, silicon nanoparticles provide more advantages in comparison with silver ones. In particular, the experimentally measured efficiency of silver nanoparticle coatings are typically lower than predicted by Fig. 4(a), which can be explained by severe degradation of silver in ambient conditions^6^,^39^. In contrast to silver, silicon has high chemical stability and low optical losses, which makes silicon nanostructures to be promising candidates for future nanophotonic devices. Moreover, recent advances in methods of their cost-effective fabrication by laser printing open up a possibility to vary nanoparticle size and crystallographic phase^34^,^40^, making silicon nanostructures even more prospective for photovoltaic applications.

The suppression of the reflection results in the resonant increase of the transmission with Si-NPC, and we define λ_*p*_ as the wavelength of maximum transmittance [Fig. 2(c)], and it is a function of nanoparticle radius *R* [Fig. 4(b)]. Specifically, the dipole resonances of silicon nanoparticles are defined by their sizes in accordance with Mie theory, and the transmittance maximum at wavelength λ_*p*_ shifts toward long wavelength region with increasing the nanoparticles sizes [Fig. 4(b)]. One can notice that the position of λ_*p*_ shifts along with the positions of ED and MD resonances staying located between them, and ED is vanished for nanoparticles smaller than 40 nm radius, and resonant enhancement is not observed [see Fig. 4(b)]. We would like to pay attention to the narrow-band increase of transmittance and introduce the peak transmission enhancement of Si-NPC as the enhancement at λ_*p*_:


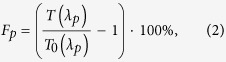


which also can be plotted as a function of nanoparticle radius (see Fig. 4(a) secondary axis of ordinate). The value of the resonant enhancement changes non-monotonically and has maximum *F*_p_ = 41% for *R* = 65 nm [see Fig. 4(a)], that relates to shifting of the resonant transmission line to the longer wavelength region, where transmittance of the bare substrate is higher.

Overall, we observe that both narrow- and broad-band transmittance increases can be obtained with nanoparticle coatings. For the increase in the entire visible range, Ag-NPC with radiuses 70–80 nm give the best transmission enhancement up to 15%, and Si-NPC with radiuses 30–40 nm causes up to 8% of enhancement. At the same time, at the narrow spectral range, Si-NPC enables up to 41% of transmittance increase.

### Antireflective coating for solar cells

High cost of energy production is one of the main problems that prevent wide implementation of solar cells^41^. For conventional planar photovoltaic structures, the thickness of crystalline silicon layer varies from 160 to 240 μm^42^. The price of materials gives a big contribution into the net costs, and decreasing the thickness of the active layer can bring a significant benefit in lowering costs. For these purposes, thin-film technology that uses amorphous silicon layers with thickness down to 150 nm is very advantageous^43^. Moreover, it has a number of valuable properties such as flexibility of the solar cells, possibility of covering functional surfaces, and other. However, comparing to conventional solar cells, thin films possess a lower capability of overall light-to-charge conversion. To improve it and increase the light absorption in the active layer, we suggest to apply the Si-NPC, and we compare their efficiency to silver plasmonic coatings^4^,^44^.

**Figure 5:**
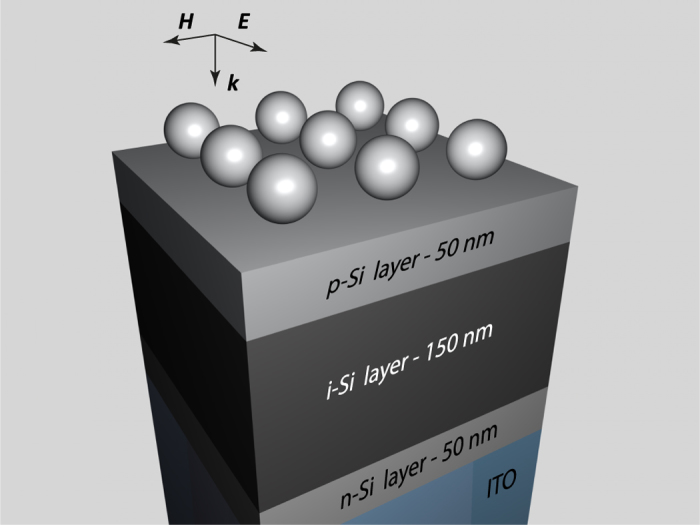
Nanoparticle coating on top of thin-film solar cells: active layer consists of 50-nm-thick p-Si, 150-nm-thick i-Si, and 50-nm-thick n-Si layers and it is on top of ITO substrate.

The typical thin film solar cell contains the active p-i-n layers for charge separating and the substrate layer (in our case indium tin oxide, ITO) (Fig. 5). For the calculations, optical parameters of the silicon were chosen with the account on different doping levels^30^, and optical parameters of ITO are taken from^45^. The absorbance in the active layer can be estimated as


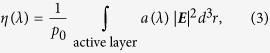


where *a*(λ) is the absorption coefficient of the active region, ***E*** is the electric field intensity inside the active region, and *P*_0_ is the incident flux density.

**Figure 6:**
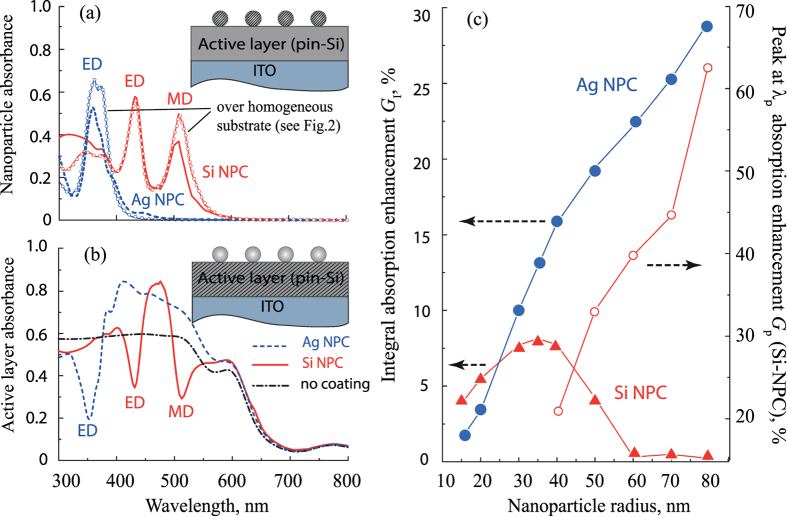
(**a**) Spectra of the light absorbance of the NPC placed over the photovoltaic element shown in Fig. 5. Lines with marks correspond to absorbance spectra of NPC placed over the homogeneous substrate [also shown in Fig. 2(a)]. Inset: schematic view of the structure; shaded region indicates where absorbance is calculated. (**b**) Absorbance inside the active layer (the region shown shaded in the Inset). (**c**) Integral absorbance enhancement for Ag-NPC and Si-NPC. The resonant absorbance enhancement at λ_*p*_ due to Kerker-type effect is also shown at secondary axis of the ordinates. The calculations in (**a**,**b**) were performed for Ag-NPC *R*_Ag_ = 30 nm, *d*_Ag_ = 125 nm and for Si-NPC *R*_Si_ = 60 nm, *d*_Si_ = 250 nm.

The results of the modeling with the proposed coatings are presented in the Fig. 6(a,b). The spectra of nanoparticle absorbance on top of the active layer [Fig. 6(a)] resemble the spectra of nanocoatings placed over the homogeneous amorphous silicon (Fig. 6(a), lines with circles) demonstrating pronounced electric and magnetic resonances. The calculated curves slightly differ from the absorbance spectra shown in Fig. 2(a) as the optical constants of doped layers differ from them for undoped silicon.

The corresponding spectra of absorbance of the active layer, shown in Fig. 6(b), is defined by the intrinsic losses in silicon, which decrease for wavelengths longer than 600 nm. The absorbance spectra of the active layer with nanoparticle coatings fully agree with spectra of light transmittance to the bulk silicon [see Fig. 2(c)]. The light absorption in the active layer at the ED and MD resonances is suppressed due to high absorption inside the nanoparticles, whereas at the wavelength of Kerker-type resonance we see a strong enhancement of light absorption. In the case of Si-NPC, the active layer absorbance possesses narrow spectral character, which reproduces the spectral behavior of enhanced transmission similar to Fig. 4.

Similar to the case of homogeneous silicon substrate [Eq. (1)], in order to compare the efficiency of different coatings we have introduced a parameter of integral absorbance enhancement in the active region, which is directly related to generated carrier current: 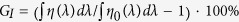 , where 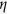 and 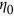 are the total absorbance in the active layer with and without coating, respectively, and the spectral integration is taken over the wavelength range from 300 nm to 800 nm. The results of the calculations are shown in the Fig. 6(c) for structures with different parameters. One can see that the integral absorption enhancement *G*_I_ has similar tendency to transmission enhancement depicted in Fig. 4(b). Particularly, the absorption efficiency of Ag-NPC grows up with increasing nanoparticle sizes and reaches 30%. The absorption enhancement *G*_I_ with Si-NPC is in agreement with the transmission enhancement *F*_I_ and has maxima of approximately 8% for nanoparticles of 30–40 nm radius. In contrast to the integral transmission enhancement shown in Fig. 4, we do not see any absorbance suppression for nanoparticles with radiuses 60–80 nm [see Fig. 6(c)] and absorption enhancement curve does not follow transmission enhancement for these radiuses. This difference can be attributed to the fact that for such big radiuses peak of transmission enhancement is placed in wavelength range, where silicon absorption is rather low. Nevertheless, the resonant absorption enhancement at the wavelength λ_*p*_ can give significantly higher values. For that we introduced the following magnitude: 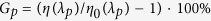, which defines the conversion enhancement calculated at the 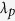. Simulation results show the monotonic increase of *G*_*p*_ with increasing of nanoparticle radius reaching the value of 65% [Fig. 6(c)]. Thus, one can see that placing NPC on top of active layer of silicon photovoltaic element can efficiently increase absorbance inside it: up to 8–10% with Ag- or Si-NPC of small radius (30–40 nm) and up to 30% with Ag-NPC of radius 80 nm having advantages of multipole resonances of plasmonic localized modes.

### Influence of protective coating on antireflective properties

Finally, we would like to address the problem of chemical stability of considered dielectric and plasmonic coatings. Nanoparticle oxidation in ambient air results in strong changes of the optical properties of the NPCs^46^,^47^,^48^. Nevertheless there are many studies where the both types of NPC (silicon and silver) are considered without taking into account their oxidizing^49^,^50^,^51^,^52^, which shows practical acceptance of such coatings. For example, silver nanoparticles placed on the top of solar cell were experimentally demonstrated to increase photocurrent up to 33%^52^. There is a variety of technological approaches to overcome oxidizing problem, reported extensively in the literature^53^,^54^,^55^,^56^,^57^. Protective coatings proposed for passivation of surface may change the optical properties of photovoltaic structures. Till now, in the present paper we have not taken any protective coating into consideration in order to keep the generality of the problem. Let us now demonstrate how typical protective coatings will influence the efficiency of the suggested NPCs and change the absorbance in the photoactive layer.

**Figure 7:**
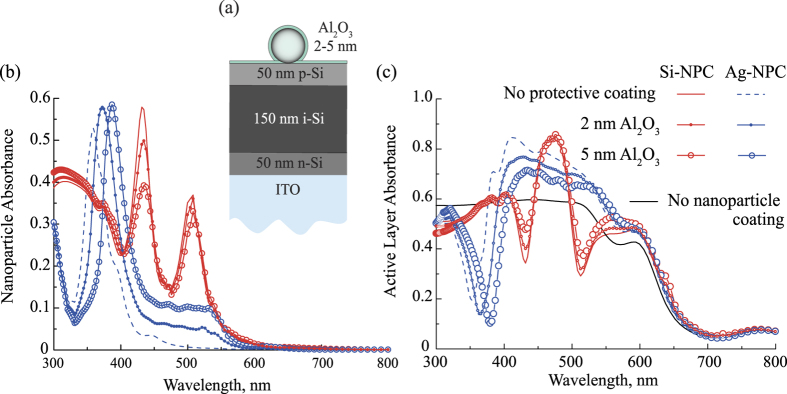
(**a**) Scheme of the structure shown in the Fig. 5 with added protective coating of Al_2_O_3_. (**b**) Spectra of the light absorbance of the NPCs placed over the photovoltaic element shown in Fig. 5 (solid line for Si-NPC and dashed line for Ag-NPC) are compared with the similar spectra for the structure shown in Fig. 7(a) (lines with scatters). (**c**) Absorbance inside the active layer shown in Fig. 5 (solid line for Si-NPC and dashed line for Ag-NPC) are compared with the similar spectra for the structure shown in Fig. 7(a) (lines with scatters). The calculations in (**b,c**) were performed for Ag-NPC *R*_Ag_ = 30 nm, *d*_Ag_ = 125 nm and for Si-NPC *R*_Si_ = 60 nm, *d*_Si_ = 250 nm.

We consider the case of protective Al_2_O_3_^58^ coating with thicknesses of 2 nm and 5 nm, which can be deposited by atomic layer deposition technique on the nanoparticle surfaces as shown in Fig. 7(a)^57^,^59^. We compare the absorption spectra without protective coating [see Fig. 6(a,b)] and with it [see Fig. 7(b,c)]. Despite there is a minor evolution of spectra, the general character of absorption spectra remains the same, and, thus, the physical interpretation of antireflectance mechanism is conserved. As one can see in Fig. 7(b), the deposition of coating results in a slight red shift of Ag-NPC plasmon resonance. Besides that, the layer of the Al_2_O_3_ between the particle and the surface gives a plateau in the absorption spectrum on the long wavelength side from the LSPR. Along with increased absorption at the resonance, this additional absorption makes the Ag-NPC with protective coating less efficient for photovoltaics applications. The inclusion of protective coating gives decreasing in the integral efficiency enhancement from 11% (without coating) to 5% and 2% integral efficiency enhancement with Al_2_O_3_ coating of 2 nm and 5 nm thickness respectively. On the contrary, the efficiency of Si-NPC coatings increases after accounting on protective coating: for the NPC parameters shown in Fig. 7 (c), the Al_2_O_3_ coating of 2 nm and 5 nm thickness gives 2% an 5% integral efficiency enhancement respectively, comparing to zero efficiency enhancement in the case of NPC without Al_2_O_3_ protection. Similar tendency is observed for other sizes of nanoparticles, and the structures showing the maximal absorbance efficiency enhancement in the active layer [see Fig. 6(c)] are of special interest. For them, for Ag-NPC (*R* = 80 nm) the integral efficiency enhancement decrease from 30% to 25 % for both 2 nm and 5 nm thicknesses of Al_2_O_3_ coating, and for Si-NPC (*R* = 35 nm) this parameter increase from 8% to 8.4 % for 2 nm and to 12.2% for 5 nm thickness of Al_2_O_3_ coating.

## Discussions

We stress that modification of directional scattering of nanoparticles near the substrate was studied before^36^ and recently gained more interest for both plasmonic^60^ and dielectric nanoparticles^35^, but none of the study addresses the problem of change of antireflective properties, in particular the important role of interference of nanoparticle multipole scattering waves with the wave reflected from the substrate. As the problem includes many parameters and the study can be performed in different ways, we did not aim to find an optimal geometry. Instead, we demonstrated a fundamental mechanism that result in anti-reflection band because of the high-index substrate layer beneath nanoparticles. In this work, we clarified nature of both anti-reflection effects, for plasmonic and high-index nanostructured coatings.

First of all, from physical point of view, Ag- and Si-NPCs differ by physical mechanisms behind their antireflective properties. In the case of substrate with positive permittivity, the localization of mode inside the silicon nanoparticle results in a weak nanoparticle-substrate interaction, which in its turn allows considering the nanoparticle array and silicon substrate as almost independent optical elements (decoupled system). In contrast, in the case of Ag-NPC, extension of mode outside nanoparticle leads to strong interaction of LSPR modes with the substrate and substrate-induced bi-anisotropy. In both cases, the antireflectance effect is related to strong light scattering by spectrally-close electric and magnetic dipoles. The main difference is that for Ag-NPC, induced magnetic dipole is effectively located at nanoparticle-substrate boundary and its magnitude substantially depends on substrate permittivity^33^, and for Si-NPC, induced magnetic dipole is inside nanoparticle and weakly affected by substrate properties. The interference of fields radiated by in-phase electric and magnetic dipole moments of nanoparticle without substrate leads to strong dominant forward scattering of light (Kerker effect^15^). In the case of silicon nanoparticles on top of high-index substrate, the antireflectance effect is observed when the dipole moments have π-phase shift, i.e. spectrally between the electric and magnetic resonances. In this sense, the suppression of the reflection, rather than suppression of backscattering from individual nanoparticle, can be assigned as a substrate-mediated Kerker effect. We need to emphasize that this effect is defined by the scattering properties of individual nanoparticles rather than collective effects related to periodicity for considered structures. Experimentally obtained single-nanoparticle spectra are in a good agreement with our simulation’s results and confirm this statement^26^. This is very important with the respect to the novel cost-effective methods of nanostructured silicon fabrication^34^,^40^.

From the practical point of view, the difference between the plasmonic and all-dielectric nanoparticle resonances results in unlikeness of integral antireflectance efficiencies [Fig. 4(b)]. For Si-NPC, it is the highest for the nanoparticles with R ≲ 35–40 nm, but becomes even negative for bigger silicon particles, whereas the integral antireflective efficiency of Ag-NPC monotonically grows. Furthermore, the antireflectance effect [summarized in Fig. 4(b)] defines properties of the coatings for enhancing thin-film photovoltaic elements efficiency [shown in Fig. 6(c)]. According to our simulations, Ag-NPC as an antireflective photovoltaic coating can be more efficiently than Si-NPC for photovoltaic application. We also performed calculations with gold nanoparticles instead of silver (not shown here) and found that for all nanoparticle sizes their efficiency is lower than for Si-NPC, because gold nanoparticles have significantly lower plasmon-resonance quality factor.

Also we should mention that the superior properties of Ag-NPC are related to the weak interaction of silicon spheres with the substrate as we discuss above, and, for instance, silicon nanodisk can be more efficiently coupled to the substrate^36^. In particular, our calculations (not presented here) show that in case of metasurfaces consisting of silicon nanodisks, the model of decoupled metasurface and high-index substrate is not valid because of the strong mode leakage from the disks to the substrate. In ref. 25. high broadband antireflectance was achieved after proper optimization of geometry of silicon disk array, and the character of this effect is not related to substrate-mediated Kerker effect revealed in the present work. Overall, the tunability of Mie resonances is one of the advantages of silicon coatings. One can speculate that the observed resonant enhancement of transmission with narrow spectral width (in a range from 20 to 70 nm) can be used in ultra-compact photodetectors and filtering systems. In a broader perspective, the studied nanoparticle arrays can be utilized in metasurfaces as a functional element in optoelectronic and photovoltaic devices.

Finally, it is worth to note that including of the thin protective coating in the considered structure leads to the minor changes of photoactive absorbance spectra. The general character of absorption spectra remains the same, and, thus, the physical interpretation of antireflectance mechanism is conserved. Numerical analysis of these spectra shows that passivating coating make Ag-NPC less efficient for using in the solar cells, while efficiency of Si-NPC increases.

## Conclusion

To summarize, we have theoretically studied silver and silicon nanoparticle arrays as antireflective coatings for high-index silicon substrate and clarified the role of magnetic moment of nanoparticles in the reflection suppression. For the silicon coatings, under illumination of visible light, electric and magnetic dipole moments are induced inside the nanoparticles. Tuning the nanoparticle radius, one can control the position of the transmittance maxima and suppression of reflection from the substrate owing to the strong dependence of dipole Mie resonances on nanoparticle’s size. The observed antireflectance with silicon coating results from destructive interference of the wave reflected from the substrate surface and light scattered by magnetic and electric dipole modes. One can consider this feature as a substrate-mediated Kerker effect, which manifests itself in zero reflection from the substrate coated with nanoparticle arrays rather than suppressed backward scattering for nanoparticles in homogeneous environment without substrate. The reflection suppression is shown for the wavelength range between the magnetic and electric dipole resonances accompanied by a resonant increase of the transmission to the substrate. For the transmission enhancement, silicon nanoparticles give up to 8% increase in a broad spectral range (300–800 nm, nanoparticles with diameters approx. 70 nm is optimal) and up to 41% peak increase (diameters approx. 130 nm is optimal). For the absorption enhancement in thin semiconductor layer, the coating gives again up to 8% increase in the same broad spectral range (diameters approx. 70 nm) and up to 64% peak increase (diameters larger than 160 nm). In the case of silver coatings, magnetic moment originates from interaction of the nanoparticle mode with the substrate, which induces “hot spot” with increased both electric and magnetic fields, referred as the substrate-induced bi-anisotropy. Similar to silicon nanoparticle array without substrate, scattering from electric and magnetic moments cancel each other resulting in zero reflection. Silver nanoparticles give up to 14% of transmission enhancement and up to 30% of enhancement of absorption in thin semiconductor layer, and nanoparticle with diameters 160 nm and more provide the best results. Thus, comparing the most optimal cases for plasmonic and all-dielectric coatings, silver nanoparticles of large size can be more efficient than the silicon coating. At the same time, the most important properties of silicon-based nanoparticle coatings are the tunability of their spectra enabled by the strong dependence of magnetic and electric resonance spectral positions on nanoparticle size, which is not the case for plasmonic nanoparticles. Moreover, according to our calculations thin 2–5 nm Al_2_O_3_ protective coating does not influence the major physical effects responsible for antireflectance, but may decrease the efficiency of silver coatings and increase the efficiency of silicon nanoparticle coating in comparison to unprotected nanoparticle coatings with the same material properties.

## Additional Information

**How to cite this article**: Baryshnikova, K. V. *et al*. Plasmonic and silicon spherical nanoparticle antireflective coatings. *Sci. Rep.* **6**, 22136; doi: 10.1038/srep22136 (2016).
